# Putting non-alcoholic fatty liver disease on the radar for primary care physicians: how well are we doing?

**DOI:** 10.1186/s12916-018-1149-9

**Published:** 2018-08-24

**Authors:** Arun J. Sanyal

**Affiliations:** 1 Division of Gastroenterology, Hepatology and Nutrition, Department of Internal Medicine, Virginia Commonwealth University, Richmond, VA USA; 2 Medicine, Physiology and Molecular Pathology, MCV Box 980341, Richmond, VA 23298-0341 USA

**Keywords:** Non-alcoholic fatty liver disease, Non-alcoholic steatohepatitis, Cirrhosis, Clinically meaningful outcomes, Burden of disease, Transient elastography

## Abstract

Non-alcoholic fatty liver disease (NAFLD) has emerged as a common cause of liver-related morbidity and mortality. Tackling this condition at a societal level will require a clear understanding of the burden of disease in the general population. However, a major limitation of such an assessment, particularly in a real-world setting, is the low rate of diagnosis of the condition, as recently identified by Alexander et al. (BMC Med 16:130, 2018). Therein, the likelihood that the condition is indeed underdiagnosed and the potential causes for such underdiagnosis are discussed. The authors underscore the need for physician education and for development of simple evaluation tools that are both robust and implementable in a primary care setting, along with effective therapeutics to overcome this apathy towards NAFLD. Importantly, there remains a need for additional data on the prevalence of non-alcoholic steatohepatitis, the more aggressive form of NAFLD, especially with progressive fibrosis, along with patient outcomes to inform health policy decisions related to screening, surveillance, and access to therapeutics.

## Background

Non-alcoholic fatty liver disease (NAFLD) has emerged as a common cause of chronic liver disease worldwide. The global prevalence estimates are concordant with estimates for obesity, type 2 diabetes, and overall caloric intake. Importantly, the number of individuals experiencing a clinically significant ‘hard’ outcome, such as death, hepatocellular cancer, or the need for liver transplant, from NAFLD is rising [1]. Based on these data, disease models project a doubling or even tripling of the number of individuals who will have end-stage liver disease by 2030 in many regions of the world [2]. These serious public health implications are driving many efforts to gain the attention of health policymakers to tackle the problem on a major ‘war-footing’.

An alternate perspective is that NAFLD is a very common condition where the majority of individuals do not have a liver-related outcome and current efforts to raise awareness may lead to ‘fear mongering’, over investigation, and even over treatment. Both perspectives appear to be reasonable; however, the question remains as to which is closer to the truth lie and how this can inform public health strategies and investment of resources for NAFLD.

In order to dissect the data to unravel the core facts several issues must be considered. First, NAFLD represents a spectrum of disease, from minor accumulation of fat without inflammation or fibrosis to severe steatosis, microscopically visible hepatocellular injury in the form of ballooning, inflammation, and pericellular fibrosis. This latter pattern is further classically seen in zone III and referred to as definite steatohepatitis, whereas intermediate forms are referred to as borderline steatohepatitis [3]. The long-term clinical outcomes are determined by the broad phenotype, i.e., non-alcoholic fatty liver (NAFL) versus non-alcoholic steatohepatitis (NASH), and the fibrosis stage [4]. While markers of disease activity have been considered irrelevant, studies making such claims have significant limitations with their internal and/or external validity. Further, recent rigorously performed studies suggest that changes in disease activity are indeed related to changes in disease progression or regression [5]. Unfortunately, it is not possible to obtain histological data in population-based studies and those defining the burden of disease rely on imaging modalities that measure steatosis, cannot distinguish between NAFL and NASH, and do not assess modest levels of fibrosis. Liver enzymes such as alanine aminotransferase (ALT) are also used as a surrogate for liver injury and, in the absence of alternate causes of liver injury such as viral hepatitis and in the presence of other risk factors for NAFLD such as obesity and type 2 diabetes, it is inferred that NAFLD is the cause of abnormal ALT.

Multiple studies have used either imaging or ALT to ascertain the prevalence of NAFLD. Of the two approaches, those that are imaging based are generally considered more accurate since a substantial proportion of individuals with NAFLD can have normal ALT. Further, of the imaging methods used, MRI is the most accurate, while sonographic evidence of an echogenic liver is only moderately sensitive and not very specific.

### Using real-world data for NAFLD management

Alexander et al. [6] report on the prevalence of NAFLD in four large healthcare systems in the UK and Europe, covering over 17 million individuals. Their study is remarkable, not only because of the large number of covered lives representative of the general population in the area, but also due to their use of ‘natural language’ and a formal ‘semantic harmonization’ approach. Through such an approach, only approximately 1% of the population was noted to carry a diagnosis of NAFLD or its subtypes NAFL or NASH.

A key question is whether these data reflect reality. Multiple studies, including population-based studies such as the Dallas Heart Study [7], where MRI-based assessment of hepatic steatosis was performed, and the Multi-Ethnic Study of Atherosclerosis (MESA) [8], have identified a higher prevalence of NAFLD. Further, using ALT as a surrogate marker of NAFLD in those without viral hepatitis or a history of heavy alcohol use, the NHANES study also identified a higher prevalence of NAFLD and increased mortality in those with elevated ALT [9]. Indeed, abundant data demonstrating a high prevalence within specific subpopulations, such as those with obesity or type 2 diabetes, would yield higher prevalence rates if extrapolated to the general population, even after accounting for ascertainment bias. It is thus likely that the authors are correct in interpreting the data to reflect under-diagnosis of the condition.

It has been reported that NAFLD is rarely looked for in the US Veterans Health Care system [10]. Indeed, even when liver enzymes are noted to be abnormal without an obvious cause, the possibility of NAFLD is not reported in the majority of individuals. Further, when a diagnosis of NAFLD is made, even lifestyle interventions are rarely provided. It is likely that the low prevalence of NAFLD noted by Alexander et al. [6] reflects a similar situation. There are several potential explanations for this lack of awareness, including gaps in education and training [11], compounded by a lack of approved therapies and limitations of the available treatments that can be used off-label. The need for a liver biopsy to confirm the diagnosis and its lack of acceptance by patients due to safety concerns, together with the technical limitations of histological assessment, represent a further barrier to the widespread evaluation and identification of affected individuals.

The study by Alexander et al. [6] further corroborates data regarding a general lack of knowledge about the care of liver disease as well as NAFLD within the physician community in a primary care setting [10], where only a minority of individuals with the diagnosis of NAFLD were assessed for liver enzyme and hepatic function, yet there was no evidence of systematic assessment of disease activity or stage. These findings highlight a need for better training in liver diseases within the primary care realm.

Nevertheless, there are some limitations to the study of Alexander et al. [6]. Firstly, it did not directly evaluate physician knowledge or attitudes regarding practice. Secondly, the potential impact of local policies related to the use of diagnostics, especially in the absence of approved therapies, is not captured. Finally, and most importantly, key data on the population of greatest interest, i.e., those with NAFLD with high disease activity and progressive fibrosis, cannot be ascertained from the study.

Together, the literature indicates that excess fat in the liver is commonly present in the general population. The challenge is to identify the subset likely to experience an adverse outcome due to such accumulation. NAFLD is associated with excess cardiovascular-, cancer-, and liver-related morbidity and mortality. However, it is unclear if regression of NAFL to a normal liver, reducing progression from NAFL to NASH, or even treatment of NASH improves the cardiovascular- and cancer-related outcomes independent of the effects of treatment on obesity and associated risk factors for these outcomes. It is therefore imperative to define the population at risk for liver outcomes; to date, stratification based on disease activity, and disease stage in particular, appears to be the best approach. A liver biopsy with histological assessment of fibrosis remains the most suitable valuation of the intermediate stages of fibrosis. However, substantial progress has been made using elastography-based methods, such as vibration-controlled transient elastography and magnetic resonance elastography, which allow accurate identification of and differentiation between those with cirrhosis and those with either none or minimal fibrosis. Through the further refinement of non-invasive tools it should become feasible to identify those most at risk of liver-related outcomes, i.e., those with active NASH with stage 2 or higher fibrosis and those with cirrhosis.

## Conclusions

The study by Alexander et al. [6] further highlights a lack of awareness of liver disease in general, and NAFLD in particular, underscoring the need for greater physician education. This is likely to be successful only through the development of simple tools that can be implemented in primary care settings to identify those at greatest risk of outcomes, along with effective therapeutics to improve outcomes in these target populations. Ultimately, common sense dictates a need for greater emphasis on healthy living and integrated preventive approaches for NAFLD and complications of the metabolic syndrome.
